# Importance of In-Hospital Prospective Registry and Infectious Endocarditis Heart Team to Monitor and Improve Quality of Care in Patients with Infectious Endocarditis

**DOI:** 10.3390/jcm10173832

**Published:** 2021-08-26

**Authors:** Guy Van Camp, Monika Beles, Martin Penicka, Dan Schelfaut, Stijn Wouters, Herbert De Raedt, Eric Wyffels, Jerrold Spapen, Riwa Nasser, Zsuzsanna Balogh, Marzia Albano, Hans De Beenhouwer, Kristien Van Vaerenbergh, Frank Van Praet, Ivan Degrieck, Bernard Stockman, Filip Casselman, Carlos Collet

**Affiliations:** 1 Cardiovascular Center, OLV Aalst, Moorselbaan 164, 9300 Aalst, Belgium; monika.beles@olvz-aalst.be (M.B.); martin.penicka@olvz-aalst.be (M.P.); dan.schelfaut@olvz-aalst.be (D.S.); Stijn.Wouters@olvz-aalst.be (S.W.); Herbert.De.Raedt@olvz-aalst.be (H.D.R.); Eric.Wyffels@olvz-aalst.be (E.W.); Jerrold.Spapen@olvz-aalst.be (J.S.); nasser.riwa@gmail.com (R.N.); Carlos.collet.bortone@olvz-aalst.be (C.C.); 2 Gottsegen Gyorgy National Institute of Cardilogy, Haller u. 29, 1096 Budapest, Hungary; zsuzsi.liliom@gmail.com; 3 Cardiology Unit, S. Maria Nuova Hospital, Via Amendola 2, 42122 Reggio Emilia, Italy; marz.albano@gmail.com; 4 Department of Microbiology, OLV Aalst, Moorselbaan 164, 9300 Aalst, Belgium; hans.de.beenhouwer@olvz-aalst.be (H.D.B.); kristien.van.vaerenbergh@olvz-aalst.be (K.V.V.); 5 Cardiovascular and Thoracic Surgery, OLV Aalst, Moorselbaan 164, 9300 Aalst, Belgium; frank.van.praet@olvz-aalst.be (F.V.P.); Ivan.Degrieck@olvz-aalst.be (I.D.); Bernard.Stockman@olvz-aalst.be (B.S.); filip.casselman@olvz-aalst.be (F.C.)

**Keywords:** infectious endocarditis, outcome, quality of care

## Abstract

Aim: To investigate the value of prospective in-hospital registry data and the impact of an infectious endocarditis heart team approach (IEHT) on improvement in quality of care and monitor outcomes in hospitalized patients with IE. Methods: Between December 2014 and the end of 2019, 160 patients were hospitalized in one centre with the definite diagnosis of infectious endocarditis (IE) and entered in a prospective registry. From 2017, an IEHT was introduced. Propensity score matching was used to assess the impact of an IEHT approach on clinical outcomes. Results: Median age was 72.5 y (62.75–80.00), diabetes was present in 33.1%, chronic kidney disease in 27.5%, COPD in 17.5%, and a history of ischaemic heart disease in 30.6%. Prosthetic valve IE was observed in 43.8% and device-related IE in 16.9% of patients. Staphylococcus (37.5%) was the most frequent pathogen followed by streptococcus (24.4%) and enterococcus (23.1%). Overall, 30-day and 1-year mortality were 19.4% and 37.5%, respectively. The introduction of prospective data collection and IE heart team was associated with a trend towards reduction of adjusted 1-year mortality (26.5% IEHT vs. 41.2% controls, *p* = 0.0699). An IEHT clinical decision-making approach was independently associated with a shorter length of stay (*p* = 0.04). Conclusions: Use of a prospective registry of IE coupled with a heart team approach was associated with more efficient patient management and a trend towards lower mortality. Prospective data collection and dedicated IEHT have the potential to improve patient care and clinical outcomes.

## 1. Introduction

Infective endocarditis (IE) is a severe disease and is still associated with high morbidity and mortality [1,2,3,4,5]. Mortality rates vary considerably between different studies. In one of the latest largest registries, the Euro-Endo Registry, including possible and definite IE, in-hospital mortality was 17.1% [1]. Landmark guidelines in the field of IE report intra-hospital mortality rates between 15–30% [2,3]. In-hospital mortality is highly influenced by the local integrated care pathway. Moreover, strategies aiming at improving care in patients with acute IE have the potential to impact mortality rates. Recent literature reports 30-day mortality of approximately 20%, whereas 1-year mortality reaches 40% [4,5].

Although the incidence of IE is low, between 3–10/100,000 per year, it increases with time despite improvements in diagnosis and treatment [6]. The complexity and heterogeneity of this disease coupled with the unabated mortality rate commits for a multidisciplinary integrated care pathway involving several specialities to pursue the best care for this fragile population. Current data on IE characteristics and impact of interventions on outcome remain scarce.

The aim of this study is (1) to demonstrate the value of in-hospital prospective data collection and an infectious endocarditis heart team approach (IEHT) decision-making pathway on the improvement in quality of care and outcomes in hospitalized patients with IE; and (2) to characterize the population of IE.

## 2. Material and Methods

### 2.1. Study Design

From December 2014, all patients hospitalized in the referral hospital OLV Aalst, Belgium, with the definite diagnosis of IE were included in a prospective registry (prespecified case report form). This registry from the Cardiovascular Centre OLV Aalst (CVCA) was created in accordance with the ethics committees of our institution. The need for written consent to participate in this research study was waived in view of its observational and anonymous nature. For this manuscript, patients entering the hospital between December 2014 and December 2019 were analysed. To be included, patients had to have the diagnosis of definite IE using the current ESC guidelines [2]. For each patient, a total of 225 variables was entered in this prospective database.

From January 2017, a multidisciplinary IEHT was organized. The IEHT was composed of cardiologists from the coronary care unit, cardiologists from the imaging and critical department, cardiac surgeons, microbiologists, neurologists, radiologists and specialists from nuclear medicine. They were all integrated in the management of the patients presenting with a suspicion of IE. From the introduction of the IEHT, all patients with a suspicion of IE were immediately admitted into the coronary care unit for close monitoring, and the IEHT was gathered the same day to decide the diagnostic and therapeutic pathways to follow for the next days, following the current ESC guidelines [2]. In the next days and weeks, patients’ clinical condition were discussed weekly in the heart team meetings. Patients were stratified into those treated following the recommendations of the IEHT and controls (i.e., patient management decided outside the structure of the IEHT before introduction of the IE heart team).

The objectives of the present study are: (1) to assess the impact of an intervention in the clinical care pathway (i.e., IEHT coupled with prospective data collection) on clinical outcomes; (2) to define patient’s characteristics and outcomes and place these results in perspective with the most recently published prospective registry data of the ESC-EORP EURO-ENDO registry and from other recent prospective registries [1,5,7,8,9].

For the evaluation of surgical indication in each case of IE, the recent ESC guidelines were used [2]. Cardiac device-related IE (CDRIE) was defined following the same ESC guidelines: an infection extending to the electrode leads, cardiac valve leaflets or endocardial surface [2]. All-cause death was assessed in-hospital, at 30 days, 6 months and 12 months. Length of stay was defined as the days from admission to discharge.

### 2.2. Data Collection and Statistical Methodology

**Figure A1:**
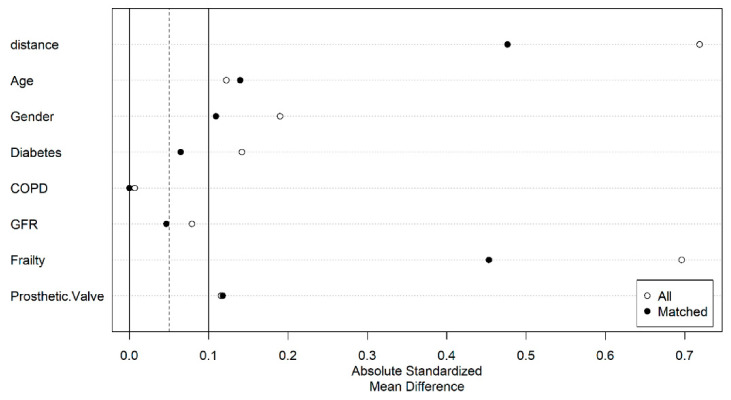
Figure with covariate balance before and after propensity score matching.

Data were collected by a dedicated research nurse (MB) in cooperation with cardiologists, cardiac surgeons and microbiologists. Results of categorical variables were represented as counts and as proportions. Continuous variables were reported as medians with interquartile range (IQR) or means and standard deviations. For comparison of observed proportions in the different groups, chi-squared test with continuity corrections were used. For among-group comparison of continuous results, unpaired t tests or Kruskal–Wallis non-parametric tests were used. In case of one sample testing of a variable on interval level, Wilcoxon rank sum test was performed. For time-to-event analysis, Kaplan–Meier survival curves were drawn. Differences in survival curves were analysed with logrank test. To compare differences in clinical outcomes between the period before and after the introduction of the registry and heart team approach, a propensity score matching was performed using a nearest neighbour. Patients were matched using 1:1 without replacement. Variables included in the propensity score were age, gender, diabetes, COPD, renal function (GFR) and presence of prosthetic valve (Figure A1). Cox regression models were used to assess independent predictors of mortality. Least square regression was used for the prediction of in-hospital length of stay. All results were considered statistically significant at *p* < 0.05 level. Data were analysed using R statistical software version 3.6.2 of the R Foundation for Statistical Computing, Vienna, Austria.

## 3. Results

A total of 160 patients with the definite diagnosis of IE were included in this study. In the same period, 27 patients had the diagnosis of possible IE and were not included in the present analysis.

### 3.1. Patient Characteristics and Comparison with Previous Registries

Patients’ characteristics are shown in Table 1. Median age was 72.5 y (62.75–80.00), diabetes was present in 33.1%, chronic kidney disease in 27.5%, COPD in 17.5%, and a history of ischaemic heart disease in 30.6%.

Patient characteristics, clinical presentation, aetiology and comparison with contemporary IE registries are shown in Table A1, Table A2 and Table A3. Staphylococcus (37.5%) was the most frequently encountered organism responsible for IE. Enterococcus, however, was almost as frequently present than streptococcus species (24.4%) in CVCA, representing 23.1% of all IE cases. This is higher compared to all other registries. From these 37 patients, 26 had a colonoscopy with resection of a polyp in 19 patients, unmasking 1 carcinoma, 2 high-grade, 3 moderate and 11 low-grade dysplasia.

**Figure A2:**
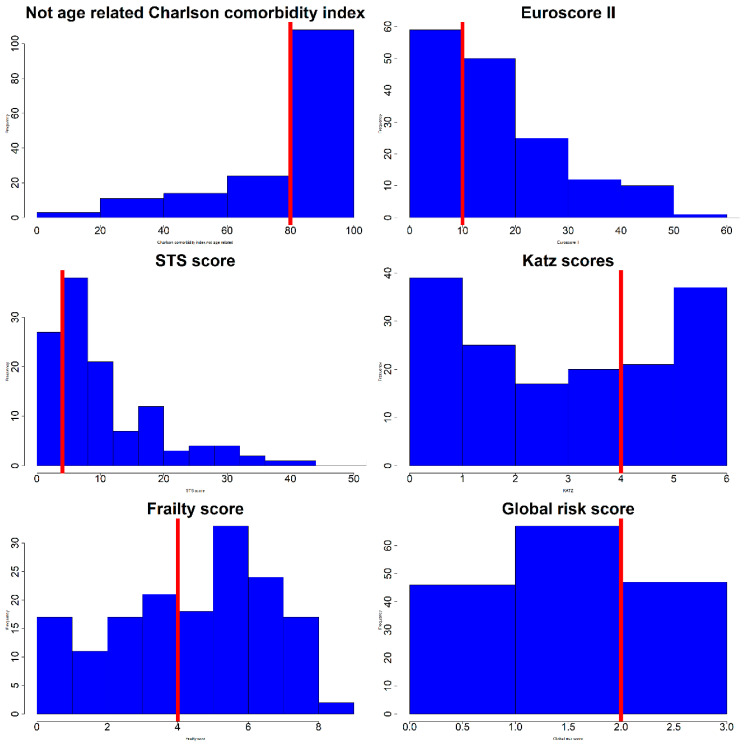
Risk scores of patients with IE from CVCA registry. Added vertical red line represents discrimination boundaries for elevated risk of mortality.

Presence of a prosthetic valve IE (46.9%) and a cardiac electronic device (27.6%) were frequently present and more frequently compared to previous reports (Table A3). Similarly, prosthetic valve IE (43.8%) and CDRIE (16.9%) were more frequently encountered compared to EURO-ENDO (30.1% and 9.9%) and other registries. (Table A3) Whatever risk estimation tool is used, the risk profile of the CVCA IE registry was high. A Charlson comorbidity index not adjusted for age ≤80 was present in 32% of IE, Euroscore II ≥10 was present in 62.5% of IE, STS score ≥4 in 60.6%, KATZ ADL <4 in 50.6%, frailty score >4 in 59% and global risk assessment was high or prohibitive in 42% and 29.3% of IE patients [3,10,11,12,13] (Figure A2, Table A4). Entrance site and acquisition of IE (Table A5) and diagnostic details from the different imaging modalities (Table A6) are shown in the Appendix A. Transthoracic echocardiography (TTE) was less often performed (53%) and transoesophageal echocardiography (TOE) more often performed compared to other registries. PET-CT was more often performed (28.8%), but MDCT was less often performed (3.1%). The sensitivity of TTE, TOE and PET-CT were comparable between CVCA and other registries with a sensitivity of TTE in CVCA of 50%, of TOE 88% and of PET-CT of 66%. Valvular lesions and complications during the course of the intra-hospital observation are also detailed in the Appendix A (Table A7 and Table A8).

### 3.2. Treatment and Clinical Outcomes

Surgery was performed in 82/160 (51.3%) patients in the CVCA registry comparable to the EURO-ENDO registry that reported 51.2% of IE patients undergoing surgery. The proportion of patients who needed surgery but in whom surgery was not performed were also comparable with 17.5% in CVCA and 18.1% in Euro-endo (*p* > 0.05). In EURO-ENDO, more patients received a mechanical prosthesis compared to CVCA (35.1% versus 3.1%; *p* < 0.001). Despite this older age, repair was performed equally in CVCA versus EURO-ENDO (11.1% versus 19.5%; *p* > 0.05) (Table 2).

**Figure 1:**
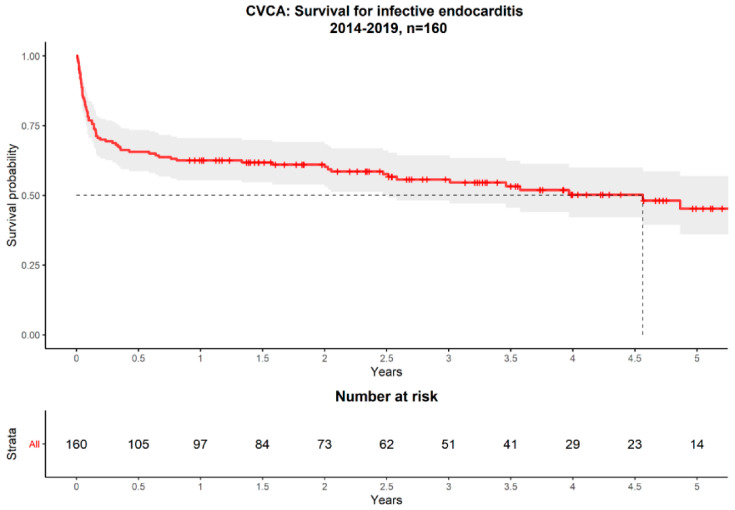
Overall survival curve of the CVCA registry 2014–2019.

Mortality at 30 days was 19.4%, at 90 days 30.6%, at 180 days 34.4% and at 1 year 37.5% (Figure 1).

**Figure 2:**
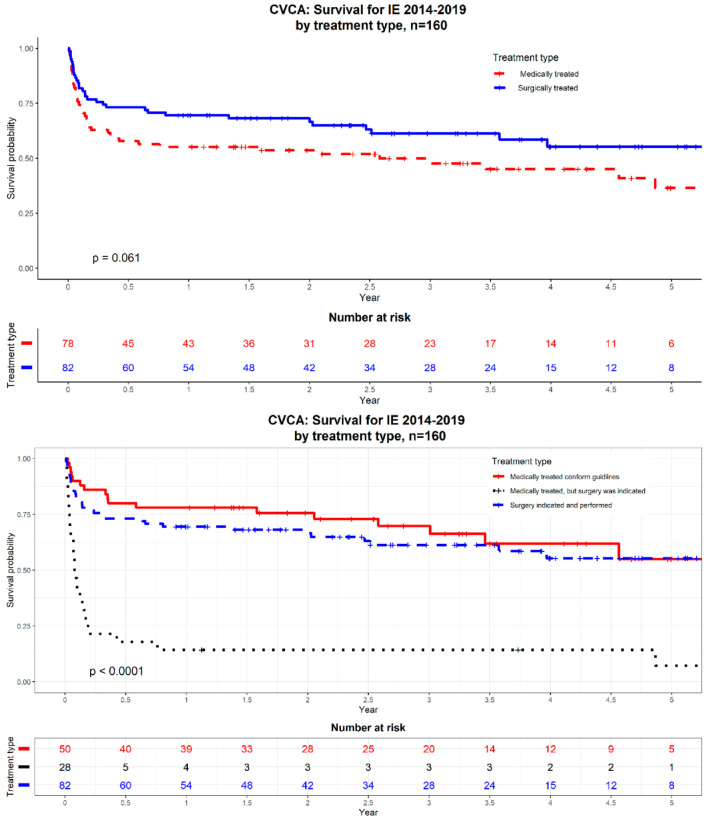
Survival curves of the CVCA registry. Panel 1: Patients grouped according medical or surgical treatment. Panel 2 patients grouped in one surgical and two medical treatment groups. Group I = medically treated patients conforming to the guidelines; Group II = surgically treated patients conforming to the guidelines; Group IIII = medically treated group who should have been operated, conforming to the guidelines, but were not operated (considered inoperable, refusal by the patient...).

In-hospital mortality was non-significantly higher in medically treated patients compared to patients undergoing surgery (Figure 2). The medically treated group was divided into a first group of patients who should have been operated conforming to the guidelines but were not (i.e., considered inoperable by the heart team or refusal by the patient or family) and a second group of patients medically treated conforming to the guidelines. Mortality in the first group was high while mortality in the second group was comparable to surgically treated patients (Figure 2, Table 3).

### 3.3. Impact of an Infective Endocarditis Heart Team and Prospective Data Collection 

Overall, 92 patients were managed following the recommendations of the IEHT and were compared with 68 controls. Baseline clinical characteristics, aetiology, risk estimation and type of IE were comparable between groups, except for frailty, which was higher in the IEHT group (5.9% vs 3.8%, *p* < 0.001) (Table 4). The observed that length of stay (LOS) tended to be shorter in patients treated following the IEHT approach (36.1 ± 25.2 days vs 44.40 ± 32 days, *p* = 0.0828). In a multivariate analysis, an IEHT decision-making approach was independently associated with a shorter LOS in hospital (−10.3 days, 95% CI: −20 to −0.5; *p* = 0.04).

**Figure 3:**
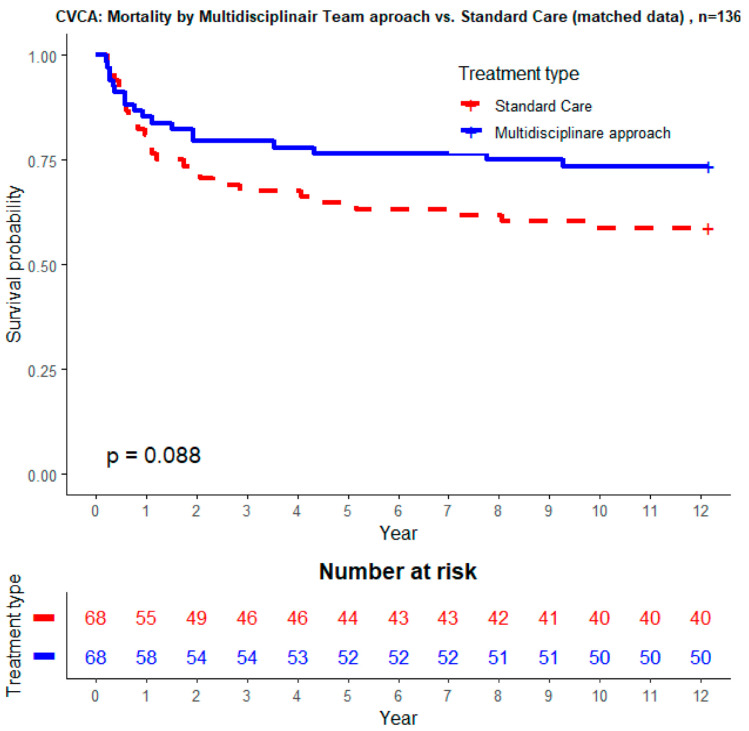
Survival curves of patients entered in the database during 2015 without IEHT compared to the survival curve of patients entered in 2019, while the IEHT was well introduced.

Table 5 shows unadjusted and adjusted mortality after propensity score, matching between patients treated under IEHT and controls. There was no difference in in-hospital mortality. However, after propensity matching, mortality at 6 months and 1 year tended to be lower in patients managed by the IEHT (23.5% IEHT vs 36.8% controls, *p* = 0.0926 and 26.5% IEHT vs. 41.2% controls, *p* = 0.0699, respectively). Figure 3 shows survival curves of patients with definite IE managed by the IEHT and controls.

## 4. Discussion

From our prospective in-hospital registry of IE, we can conclude that compared with contemporary literature, our population with IE: 1) is at higher risk, has more enterococcus IE probably related to older age, has more PVE and CDRIE, is detected with similar sensitivity compared to other centres by echo and PET, is as frequently operated, has a 30-day and 1-year mortality comparable with mortality rates mentioned in current literature. Mortality was high in those patients treated medically, although surgery was theoretically indicated. 2) The introduction of IEHT led to a reduction in LOS with a trend towards reduction in mortality. These results demonstrate that an in-hospital registry for IE and a multidisciplinary approach can be used as a management tool to evaluate and improve the quality of care of patients with IE.

Benchmarking medical management of every disease starts with precise characterization of the population studied. These characteristics of patients with IE can vary depending on several hospital and environmental aspects. Comparison of our population with the most recent published registries illustrate that our patients were older, had more frequent diabetes, COPD, chronic kidney disease and prosthetic valves or electronic devices [1,5,7,8,9]. This resulted in a high-risk population documented by high-risk scores (i.e., STS, Euroscore II, Charlson co-morbidity, Katz score, frailty and global risk score). All the patient characteristics associated with poor outcome mentioned in the latest guidelines were more frequently present in the CVCA registry: older age, prosthetic valve IE, diabetes mellitus, comorbidity (e.g., frailty, renal, or pulmonary disease) [2]. These high-risk characteristics must be acknowledged in the comparison of outcome with other registries.

The microbiological findings of our IE population are mostly in line with current literature [14,15,16,17]. In developed countries, IE patients are older and IE cases are more frequently related to health-care facilities. Prosthetic valves and CIED devices have replaced rheumatic disease as the main risk factor for IE [2,14,15,16]. In parallel, staphylococcus has replaced streptococcus as the most frequently isolated pathogen.

Staphylococcus aureus is also in CVCA, the most frequently encountered organism responsible for 37.5% of IE, followed by Streptococcus IE (24.4%). Enterococcus, however, is almost as frequently present as Streptococcus species (23.1%). This is higher compared to all other registries. This can probably be explained by the older age of the CVCA registry and is illustrated by the frequent finding of a digestive entrance place (multiple polyps and cancer). In CVCA registry, IE was frequently health care related (40%) with an important representation of non-nosocomial health care related IE that can be explained by the old age of this population living frequently in residential care centres.

PVE (43.8%) and CDRIE (16.9%) represent a larger group of IE patients in the CVCA registry compared to other current registries [1,5,7,8,9]. This can be explained by the fact that within a developed country, the hospital is a referral centre for valvular heart disease. Patients that were operated on (valve surgery) or had device implants in the hospital are also referred to the CVCA when complications occur. This aspect must be considered when evaluating the outcome of IE patients since mortality is higher in PVE and CDRIE compared to native valve IE.

TTE was less often performed in the CVCA registry, which is due to the fact that TTE is often already performed by the referral hospital and exams are available when the patients arrive in the CVCA. TEE and PET-CT were frequently performed and the more frequent use of PET-CT in the CVCA registry results probably also in the less frequently performed MDCT. The diagnostic yield of these imaging techniques was comparable with other registries. Since not one of these techniques is the holy grail, multi-modality imaging is key in the diagnosis of IE [18].

The CVCA registry counts less culture negative IE (3.8%), illustrating the high performance of microbiological detection of IE. The microbiological team is active in the hospital, working with the different departments when blood cultures are positive, while sensible physicians actively perform complementary imaging techniques or perform supplementary blood cultures in patients with unexplained infections.

Complications are similar in CVCA and other current registries [1,5,7,8,9]. Surgery was performed in 82/160 (51.3%) patients in the CVCA registry, also comparable with the other registries. The proportion of patients who needed surgery but in whom surgery was not performed (18.1%) is also comparable. Less mechanical valves are implanted in the CVCA registry, probably linked to older age. Despite this older population, valve repair was performed as frequently as in the recent EURO-ENDO registry, illustrating the efforts of the surgical department to repair valves in IE when possible.

Mortality of IE in CVCA in-hospital was 28.7%, at 30 days 19.4%, at 90 days 30.6%, at 180 days 34.4% and at 1 year 37.5%. These results are consistent with current literature. In-hospital mortality is difficult to compare between studies and registries since it depends on the regional health care organization (early versus late discharge; early versus late referral to other care facilities). Therefore, 30-day and 1-year mortality should also be used as reliable metrics. The 30-day mortality of 19.4% was comparable, with most publications reporting mortality rates of 10–30% [4,19]. A 1-year mortality of 37.5% is also comparable with literature, reporting rates of 21–40% [5,8,9,18]. CVCA mortality rate cannot be compared with the EURO-ENDO registry since only in-hospital mortality was reported (17.1%). The 1-month mortality is missing in the other registries but the 1-year mortality of the RIEI registry was 16.7% and 37.8% in the study of Hidalgo et al [8,9]. Comparison of outcomes of IE between prospective studies and registries remains difficult, but considering the high-risk profile of the CVCA population and the inclusion of only definite IE cases (EURO-ENDO [1] and Hidalgo et al. [9] included possible and definite IE), outcome in the CVCA registry compares favourably with other data.

Patients treated medically or surgically following the guidelines have the same favourable outcome. Contrarily, patients treated medically, although the guidelines indicate surgical treatment, have a dramatic high mortality. This finding is consistent with the EURO-ENDO registry [1].

Linked to the high mortality of IE and the multi-system involvement in most patients with IE, current guidelines encourage an organized multidisciplinary approach for these patients, including cardiologists, cardiac surgeons, cardiac anaesthetists, neurologists, microbiologists and intensive care physicians and the use of clinical pathways integrating the existing guidelines [2,19,20]. Although the heart team has already functioned many years in the hospital, we organized an IEHT in 2017, implementing a multidisciplinary approach with a focus on early recognition with acute close monitoring in the cardiac intensive care unit, early multidisciplinary decision-making using current guidelines and close follow-up in the heart team during the course of the hospitalization. The prospective registry enables us to compare the current outcome of patients with IE before the introduction of the IEHT (year 2014–2016) with the period with IEHT (2017–2019). We found an important reduction of the LOS of more than 8 days. An IEHT decision-making approach was independently associated with a shorter length of stay in-hospital. This can be explained by the early decision making within the IEHT. We observed a tendency towards a better outcome with IEHT (1-year mortality decreases from 41.2% to 34.8%) despite a higher frailty in the IEHT group. After propensity score matching, 1-year mortality decreased from 41.2% before IEHT to 26.5% with IEHT (*p* = 0.0699), illustrating, besides the impact on the LOS, the potential benefit of an early multidisciplinary approach on outcomes.

Future perspectives: Considering the great proportion of PVE and CDRIE in our CVCA registry, prophylactic measurements to prevent this deadly disease should be encouraged. Supplementary tools to instruct our patients with prosthetic valves and electronic devices to follow rigorously prophylactic measures against IE will be introduced. The multidisciplinary approach will be continued within the IEHT with a special attention to the group of patients in whom surgery would be denied in contradiction with the guidelines. This will be challenging due to the older age and the frailty of our population. Finally, patients will be followed in the valve clinic after hospital discharge, knowing the still high mortality in this population.

## 5. Limitations

The major limitation of this single-centre study is the low number of patients. However, a complete dataset of current prospective obtained data in 160 patients with long-term follow-up enhances the knowledge in the domain of IE in which data are still scarce.

## 6. Conclusions

We can conclude from our CVCA registry that in-hospital prospective registration of data in IE patients permits to characterize the particular IE patient population of the hospital, which is a high-risk population in CVCA and which can differ significantly from other published registries. These differences can have a major impact on the interpretation of the outcome. In-hospital prospective registration of IE patients is the only way to monitor the effect of interventions to lower mortality in this disease. Implementation of IEHT resulted in an important decrease in the LOS with a non-significant trend for lower mortality.

## Figures and Tables

**Table 1:** Patient characteristics.

Registry	CVCA
*n* hospitals	1
*n* countries	1
Data collection period	2014–2019
Cases included	Definite IE
***Demography:***	
N	160
Median age in years (IQR)	72.50 (62.75 ± 80.00)
Age ≥ 65 years	112/160 (70%)
Age ≥ 80 years	44/160 (27.5%)
Females	40/160 (25%)
***History of cardiovascular diseases:***	
Heart failure	33/160 (20.6%)
Congenital disease	13/160 (8.1%)
Ischaemic heart disease	49/160 (30.6%)
Peripheral arterial disease	28/160 (17.5%)
Previous endocarditis	15/160 (9.4%)
Intra-cardiac device	44/160 (27.6%)
Presence of prosthetic valve	75/160 (46.9%)
***Risk factors and comorbidities:***	
Diabetes	53/160 (33.1%)
Previous stroke/TIA	14/160 (8.8%)
COPD/asthma	28/160 (17.5%)
Chronic renal failure	44/160 (27.5%)
Dialysis	19/160 (11.9%)
HIV	2/160 (1.2%)
Chronic liver disease	13/160 (8.2%)
Cancer	27/160 (16.9%)
Intravenous drug dependency	3/160 (1.9%)
Intravenous catheter	12/160 (7.5%)
Invasive procedure within 60 days	43/160 (26.9%)

Overview of basic study specifications, demography, cardiovascular disease history, risk factors and comorbidities in the CVCA.

**Table 2:** Treatment.

Registry	CVCA	EURO-ENDO	ICE-PCS	RIEI	Fernández-Hidalgo et al. Hospital Universitari Vall d’Hebron, Spain
***Treatment type:***					
Indication for surgery	110/160 (68.8%)	2160/3115 (69.3%)			318/438 (72.6%)
Surgery performed	82/160 (51.3%)	1596/3116 (51.2%)	1335/2769 (48.0%)	422/677 (62.3%) *	174/438 (39.7%) *
***Type of surgery performed:***					
Mechanical prosthesis	5/81 (3.1%)	560/1596 (35.1%) ***			135/438 (30.8%) ***
Bioprosthesis	61/81 (75.3%)	817/1596 (51.2%) ***			36/438 (8.2%) ***
Repair	9/81 (11.1%)	311/1596 (19.5%)			3/438 (0.6%) ***
Surgery indicated but not performed	28/160 (17.5%)	564/3115 (18.1%)			144/438 (32.8%) ***
Medical treatment (surgery not indicated)	50/160 (31.3%)	955/3115 (30.6%)			161/438 (36.8%)

Comparison of the IE treatment in the five studies. Cells markings with asterisks represents the statistical significance levels of the two-sample Pearson’s chi-squared tests for the observed proportions. Reported significance levels for pairwise comparison between OLV Hospital and each of the four other registries individually are the following: No asterisk marking given for *p* >= 0.05, * for *p* < 0.05 and *** for *p* < 0.001.

**Table 3:** Risk and outcome.

Registry	OLV Hospital	EURO-ENDO	ICE-PCS	RIEI	Fernández-Hidalgo et al. Hospital Universitari Vall d’Hebron, Spain
***Risk assessments:***					
Median EuroSCORE	13.3 (6.2–24.0)	5.0 (2.0–13.3) ***			
Median EuroSCORE when surgery performed (IQR)	12.5 (6.2–22.3)				9 (9–12) ***
Median EuroSCORE when surgery indicated but not performed (IQR)	17.8 (9.5–26.7)				11 (8–13) **
Subjects with definite Modified Duke criteria	160	2610/3116 (83.8%) ***			386/438 (88.1%) ***
Median EuroSCORE prosthetic IE (IQR)	19.0 (11.1–27.5)	10.9 (5.2–24.9) ***			
Median EuroSCORE native IE (IQR)	8.6 (3.2–18.8)	3.0 (1.5–8.0) ***			
Median EuroSCORE device-related IE (IQR)	9.0 (5.2–16.3)	6.0 (2.2–15.0) **			
***Mortality:***					
In-hospital mortality	46/160 (28.7%)	532/3116 (17.1%) ***	490/2774 (18.0%) ***	93/677 (13.7%) ***	125/677 (28.5%)
In-hospital mortality prosthetic IE	18/70 (25.7%)	187/939 (19.9%)			
In-hospital mortality native IE	19/63 (30.2%)	286/1764 (16.2%) **			
In-hospital mortality device-related IE	9/27 (30%)	47/308 (15.3%) *			
Cumulative 1-month mortality	31/160 (19.4%)				
Cumulative 3-month mortality	49/160 (30.6%)			(13.8%) ***	
Cumulative 6-months mortality	55/160 (34.4%)			(15.5%) ***	
Cumulative 1-year mortality	60/160 (37.5%)			(16.7%) ***	150/397 (37.8%)

Overview of risk assessment and mortality outcome reported in the five studies. Cells markings with asterisks represents the statistical significance levels of the two-sample Pearson’s chi-squared tests for the observed proportions and of the one-sample Wilcoxon rank sum tests for the medians. Reported significance levels for pairwise comparison between OLV Hospital and each of the four other registries individually are the following: No asterisks marking given for *p ≥* 0.05, * for *p* < 0.05, ** for *p* < 0.01 and *** for *p* < 0.001.

**Table 4:** Baseline clinical characteristics, aetiology, risk assessment and type of IE: IEHT approach versus standard care.

	IEHT Approach	Standard Care	*p* Value
***Baseline characteristics***			
Age	6/69.5 (sd: 13.3)	71.0 (sd: 11.8)	0.4710
Gender (male)	66/92 (71.7%)	54/68 (79.4%)	0.3558
Diabetes	33/92 (35.9%)	20/68 (29.4%)	0.4914
COPD	16/92 (17.6%)	12/68 (17.4%)	0.9999
GFR	52.1 (sd: 28.7)	54.1 (sd: 25.7)	0.6393
Previous cardiac surgery	44/92 (47.8%)	36/68 (52.9%)	0.6314
Previous coronary artery disease	25/92 (27.2%)	24/68 (35.3%)	0.3534
Stroke	8/92 (8.7%)	6/68 (8.8%)	0.9999
Peripheral arterial disease	13/92 (14.1%)	15/68 (22.1%)	0.2738
LVEF < 50%	23/92 (25.0%)	10/68 (14.7%)	0.1835
Degenerative Valve Disease	54/92 (58.7%)	35/68 (51.5%)	0.4542
Previous endocarditis	9/92 (9.8%)	6/68 (8.8%)	0.9999
Cancer	14/92 (19.1%)	13/68 (15.2%)	0.6616
***Aetiology***			
Streptococcus viridans	8/92 (8.7%)	9/68 (13.2%)	0.5082
Staphylococcus aureus	27/92 (29.4%)	20/68 (29.3%)	0.9999
Enterococcus	18/92 (19.6%)	19/68 (27.9%)	0.2925
***Risk Assessment***			
Charlson index (not age related)	82.7 (sd: 21.4)	81.1 (sd: 27.3)	0.6745
Frailty score	5.6 (sd: 1.8)	3.8 (sd: 2.5)	0.0001
Euroscore II	16.7 (sd: 12.6)	17.4 (sd: 16.7)	0.7535
KATZ score	3.2 (sd: 2.1)	3.4 (sd: 2.3)	0.6216
Global Risk score	1.8 (sd: 0.8)	2.1 (sd: 0.9)	0.0879
***IE Type:***			
Prosthetic	38/92 (41.3%)	32/68 (47.1%)	0.1610
Native	34/92 (37.0%)	29/68 (42.6%)	
Device-related	20/92 (21.7%)	7/68 (10.3%)	

Overview of basic characteristics, demography, cardiovascular disease history, risk factors and comorbidities before propensity score matching: IEHT approach versus standard care.

**Table 5:** Outcome IEHT approach versus standard care.

	IEHT Approach	Standard Care	*p* Value
Unadjusted mortality			
In-hospital mortality	28.3%	29.4%	0.8737
30 days mortality	20.7%	20.7%	0.8104
180 days mortality	32.6%	36.8%	0.5843
1 year mortality	34.8%	41.2%	0.4089
After propensity score matching			
In-hospital mortality	17.7%	29.4%	0.1058
30 days mortality	14.7%	19.1%	0.4926
180 days mortality	23.5%	36.8%	0.0926
1 year mortality	26.5%	41.2%	0.0699

Comparison of mortality data In-hospital, after 30 days, after 180 days, after 1 year between IEHT approach and standard care unadjusted and after propensity score matching.

## Data Availability

The data underlying this article are available in the article.

## Appendix A

**Table A1:** Patient characteristics (CVCA and other registries).

Registry	CVCA	EURO-ENDO	ICE-PCS	RIEI	Fernández-Hidalgo et al. Hospital Universitari Vall d’Hebron, Spain
N hospitals	1	156	58	17	1
N countries	1	40	25	1	1
Data collection period	2014–2019	January 2016–March 2018	2000–2005	2007–2010	2000–2011
Cases included	Definite IE	Possible with treatment & Definite IE	Definite IE	Definite IE	Left-sided possible and definite IE
***Demography:***					
N	160	3116	2781	677	438
Median age in years (IQR)	72.50 (62.75 ± 80.00)	63.0 (46.0–73.0) ***	57.9 (43.2–71.8) ***	65.34 (49.60–74.39) ***	66.4 (51.8–74.9) ***
Age ≥ 65 years	112/160 (70%)	1443/3116 (46.3%) ***			
Age ≥ 80 years	44/160 (27.5%)	375/3116 (12.0%) ***		60/677 (8.7%) ***	
Females	40/160 (25%)	969/3116 (31.1%)	888/2777 (32.0%)	185/677 (27.3%)	153/438 (34.9%) *
***History of cardiovascular diseases:***					
Heart failure	33/160 (20.6%)	662/2840 (23.3%)			
Congenital disease	13/160 (8.1%)	365/3114 (11.7%)	311/2656 (12.0%)	39/677 (5.8%)	
Ischaemic heart disease	49/160 (30.6%)	622/2897 (21.5%) **		95/677 (14.0%) ***	
Peripheral arterial disease	28/160 (17.5%)				
Previous endocarditis	15/160 (9.4%)	274/3116 (8.8%)	222/2780 (8.0%)		
Intra-cardiac device	44/160 (27.6%)	537/3116 (17.2%) **		122/677 (18.0%) **	
Presence of prosthetic valve	75/160 (46.9%)		618/2686 (23%) ***	178/677 (26.3%) ***	
***Risk factors and comorbidities:***					
Diabetes	53/160 (33.1%)		447/2764 (16.0%) ***	116/677 (17.1%) ***	99/438 (22.6%) *
Previous stroke/TIA	14/160 (8.8%)	340/2860 (11.9%)		35/677a (5.2%)	
COPD/asthma	28/160 (17.5%)	318/3111 (10.2%) **		65/677 (9.6%) **	
Chronic renal failure	44/160 (27.5%)	553/3113 (17.8%) **		85/677 (12.6%) ***	
Dialysis	19/160 (11.9%)	163/3113 (5.2%) ***	220/2777 (8.0%)		22/438 (5.0%) **
HIV	2/160 (1.2%)	31/3038 (1.0%)	58/2748 (2.0%)	40/677 (5.9%) *	15/438 (3.4%)
Chronic liver disease	13/160 (8.2%)			64/677 (9.5%)	
Cancer	27/160 (16.9%)	361/3088 (11.7%)	230/2772 (8.0%) ***	66/677 (9.8%) *	56/438 (13.5%)
Intravenous drug dependency	3/160 (1.9%)	212/3067 (6.9%) *	268/2746 (10.0%) **	61/677 (9.0%) **	12/438 (2.7%)
Intravenous catheter	12/160 (7.5%)	250/3104 (8.1%)	244/2763 (9.0%)	71/677 (10.5%)	
Invasive procedure within 60 days	43/160 (26.9%)		690/2581 (27.0%)	171/677 (25.3%)	

Overview of basic study specifications, demography, cardiovascular disease history, risk factors and comorbidities in the five samples. Cell markings with asterisks represent the statistical significance levels of the two-sample Pearson’s chi-squared tests for the observed proportions and of the one-sample Wilcoxon rank sum tests for the medians. Reported significance levels for pairwise comparison between OLV Hospital and each of the four other registries individually are the following: no asterisks marking given for *p* >= 0.05, * for *p* < 0.05, ** for *p* < 0.01 and *** for *p* < 0.001.

**Table A2:** Clinical presentation.

Registry	CVCA	EURO-ENDO	ICE-PCS	RIEI	Fernández-Hidalgo et al. Hospital Universitari Vall d’Hebron, Spain
***Clinical onset and assessment:***					
First sign to admission < 1 month	141/160 (88.1%)		2088/2711 (77.0%)	340/677 (50.2%) ***	
Transferred from other centres	45/160 (28.1%)			305/677 (45.0%) ***	170/438 (38.8%) *
Fever	95/160 (59.4%)	2383/3068 (77.7%) ***	2322/2428 (96.0%) ***	568/677 (83.9%) ***	
Increased C-reactive protein	159/160 (99.4%)		1632/2650 (62.0%) ***	566/877 (83.6%) ***	
New murmur or worsening old murmur	40/160 (25%)	2008/3112 (64.5%) ***	1970/2781 (70.8%) ***	358/677 (52.9%) ***	
Haematuria	69/160 (43.1%)		666/2587 (26.0%) ***	97/677 (14.3%) ***	
Creatinine > 2	45/160 (28.1%)			75/677 (11.1%) ***	
Osler nodes	1/160 (0.6%)	60/3116 (1.9%)	77/2648 (3.0%)	26/677 (3.8%)	
Splint haemorrhage	1/160 (0.6%)		213/2655 (8.0%) **	9/677 (1.3%)	
Janeway lesion	2/160 (1.2%)	109/3116 (3.5%)	123/2650 (5.0%)	6/677 (0.9%)	
Conjunctival haemorrhage	2/160 (1.2%)		122/2655 (5.0%)	6/677 (0.9%)	
Roth spots	2/160 (1.2%)	44/3052 (1.4%)	50/2649 (1.89%)		

Summary of the clinical onset and assessments reported in the five studies. Cell markings with asterisks represent the statistical significance levels of the two-sample Pearson’s chi-squared tests for the observed proportions. Reported significance levels for pairwise comparison between OLV Hospital and each of the four other registries individually are the following: no asterisks marking given for *p* >= 0.05, * for *p* < 0.05, ** for *p* < 0.01 and *** for *p* < 0.001.

**Table A3:** Type and etiology of IE.

Registry	CVCA	EURO-ENDO	ICE-PCS	RIEI	Fernández-Hidalgo et al. Hospital Universitari Vall d’Hebron, Spain
***Type IE:***					
Native valve	63/160 (39.4%)	1764/3116 (56.6%) ***	1901/2636 (72.0%) ***	445/677 (65.7%) ***	337/485 (76.9%) ***
Prosthetic valve	70/160 (43.8%)	939/3116 (30.1%) ***	563/2636 (21.0%) ***	128/677 (18.9%) ***	101/485 (23.1%) ***
Intra-cardiac device related	27/160 (16.9%)	308/3116 (9.9%) **	172/2636 (7.0%) ***	94/677 (13.9%)	
***Aetiology:***					
Positive blood culture	154/160 (96.3%)	2461/3116 (79.0%) ***		492/677 (72.7%) ***	
Streptococci	39/160 (24.4%)				163/438 (37.2%) **
Viridans streptococci	17/160 (10.6%)	304/2461 (12.4%)	483/2781 (17.0%) *	73/677 (14.8%)	103/438 (23.5%) ***
Streptococcus bovis complex	12/160 (7.5%)		165/2781 (6.0%)	57/677 (11.6%)	33/438 (7.5%)
Streptococcus agalactiae	3/160 (1.9%)				
Pneumococcus	5/160 (3.1%)				
Other streptococci	1/160 (0.6%)		162/2781 (6.0%) **		
Staphylococci	60/160 (37.5%)	1085/2461 (44.1%)			143/438 (32.6%)
Staphylococcus aureus	47/160 (29.4%)	772/2461 (31.3%)	869/2781 (31.0%)	133/677 (27.0%) **	99/438 (22.6%)
Methicillin-resistant S. aureus	0/160 (0%)	177/2461 (7.2%) ***		37/677 (5.4%) **	23/438(5.3%) **
Coagulase-negative staphylococci	13/160 (8.1%)	313/2461 (12.7%)	304/2781 (11.0%)	106/677 (21.6%) *	44/438 (10.0%)
Enterococci	37/160 (23.1%)	390/2461 (15.8%) *	283/2781 (10.0%) ***	75/677 (15.1%) ***	59/438 (13.5%) **
Gram-negative rods (GNR)	3/160 (1.9%)	86/2461 (3.5%)			19/438 (4.3%)
HACEK group	1/160 (0.6%)		44/2781 (2.0%)	6/677 (1.2%)	9/438 (2.1%)
Fungi/yeast	1/160 (0.6%)		45/2781 (2.0%)		
Polymicrobial	4/160 (2.5%)		28/2781 (1.0%)		
Culture negative	6/160 (3.8%)		310/2781 (11.1%) **		

Overview of the infective endocarditis type and etiology reported in the five studies. Cell markings with asterisks represent the statistical significance levels of the two-sample Pearson’s chi-squared tests for the observed proportions. Reported significance levels for pairwise comparison between OLV Hospital and each of the five other registries individually are the following: No asterisk marking given for *p* >= 0.05, * for *p* < 0.05, ** for *p* < 0.01 and *** for *p* < 0.001.

**Table A4:** Risk scores in CVCA registry.

	Prosthetic IE *n* = 70	Native IE *n* = 63	Cardiac Device Related IE *n* = 27	Significance Level of *p*-Value	Overall *n* = 160
Median EuroSCORE (IQR)	19.0 (11.1–27.5)	8.6 (3.2–18.8)	9.0 (5.2–16.3)	***	13.3 (6.2–24.0)
Median STS score *(only calculated for aortic or mitral valve surgeries)*	8.6 (5.7–17.8)	6.6 (3.8–13.5)	6.8 (4.8–24.6)		7.5 (4.2–15.9)
Global risk score >= 2	49 (70.0%)	44 (69.8%)	21 (77.8%)		114 (71.3%)
Median Charlson comorbidity index-not age related (IQR)	96.0 (90.2–98.3)	90.2 (77.5–98.3)	90.2 (53.4–95.9)	*	90.2 (77.4–98.3)
Median Katz score (IQR)	4.0 (2.0–5.0)	3.0 (1.0–5.0)	4.0 (2.0–5.8)		3.0 (2.0–5.0)
Median frailty score (IQR)	4.5 (3.0–6.0)	6.0 (4.0–7.0)	5.0 (4.0–6.0)		5.0 (3.0–7.0)

Risk score in the different groups of IE. Median values are shown. No asterisks marking given for *p* >= 0.05, * for *p* < 0.05 and *** for *p* < 0.001.

**Table A5:** Entrance site and acquisition.

Registry	CVCA	EURO-ENDO	ICE-PCS	RIEI	Fernández-Hidalgo et al. Hospital Universitari Vall d’Hebron, Spain
***Entrance site:***					
Oral/dental	16/160 (10%)	(9.8%)			
Digestive	39/160 (24.4%)	(6.3%) ***			
Genitourinary	7/160 (4.4%)	(4.5%)			
Skin	33/160 (20.6%)				
***Acquisition:***					
Community-acquired	92/160 (57.5%)	2046/3116 (65.7%) *	1975/2781 (71.0%) ***	425/677 (62.8%)	
Healthcare related	64/160 (40%)	1027/3116 (33.0%)	640/2781 (23%) ***	160/677 (23.6%) ***	135/438 (30.8%) *
Nosocomial	15/160 (9.4%)	624/3116 (19.5%) **	390/2781 (14.0%)	76/677 (11.2%)	
No-nosocomial healthcare related	49/160 (30.6%)	403/3116 (12.9%) ***	250/2781 (9.0%) ***	84/677 (12.4%) ***	
Unknown	4/160 (2.5%)	43/3116 (1.4%)	167/2781 (6.0%)	92/677 (13.6%) ***	

Comparison of the IE entrance site and acquisition reported in the five studies. Cell markings with asterisks represent the statistical significance levels of the two-sample Pearson’s chi-squared tests for the observed proportions. Reported significance levels for pairwise comparison between CVCA and other registries. No asterisks marking given for *p* >= 0.05, * for *p* < 0.05, ** for *p* < 0.01 and *** for *p* < 0.001.

**Table A6:** Diagnostic Imaging.

Registry	CVCA	EURO-ENDO	ICE-PCS	RIEI	Fernández-Hidalgo et al. Hospital Universitari Vall d’Hebron, Spain
**Clinical imaging**					
Cardiac echo performed	151/160 (94.4%)	3111/3116 (99.8%) ***			
TTE performed	85/160 (53%)	2793/3111 (89.8%) ***		640/677 (94.5%) ***	
TEE performed	148/160 (92.5%)	1808/3111 (58.1%) ***		478/677 (70.6%) ***	
TTE positive	42/85 (49.4%)			395/640 (62.0%)	
TEE positive	130/148 (87.8%)			447/478 (93.5%) ***	
PET CT performed	46/160 (28.8%)	518/3116 (16.6%) ***			
Leucocyte scintigraphy	1/160 (0.6%)	38/3116 (1.2%)			
MDCT	5/160 (3.1%)	1656/3116 (53.1%) ***			

Clinical imaging in the five studies. Cell markings with asterisks represent the statistical significance levels of the two-sample Pearson’s chi-squared tests for the observed proportions. Reported significance levels for pairwise comparison between OLV Hospital and each of the four other registries individually are the following: no asterisk marking given for *p* >= 0.05 and *** for *p* < 0.001.

**Table A7:** Valvular lesions in CVCA registry.

	Aortic Valve	Mitral Valve	Tricuspid Valve	Pulmonal Valve
Prosthetic	56	22	3	0
Vegetation	66/160 (41.3%)	55/160 (34.4%)	14/160 (8.8%)	1/160 (0.6%)
Vegetation median size in mm (IQR)	10.0 (7.0–13.0)	12.5 (9.0–20.0)	16.0 (10.0–20.0)	18 (18.0–18.0)
Vegetation range in mm	2–35	3–50	6–25	–
Valve regurgitation >= 3/4	33	27	16	1
Leaflet perforation	9	17	3	0
Periannular abscess	28	5	0	0
Periannular pseudoaneurysm	3	2	0	0
Prosthetic valve dehiscence	12	4	1	0
Prosthetic valve obstruction	2	0	0	0

CVCA diagnostic imaging results.

**Table A8:** Complications.

Registry	CVCA	EURO-ENDO	ICE-PCS	RIEI	Fernández-Hidalgo et al. Hospital Universitari Vall d’Hebron, Spain
***Complications (at admission or while under treatment):***					
Congestive heart failure	69/160 (43.1%)	1288/3116 (41.3%)	876/2713 (32.0%) **	301/677 (44.5%)	205/438 (46.8%)
Intracardiac abscess	31/160 (19.4%)	556/3116 (17.8%)	389/2707 (14.0%)	77/677 (11.4%) **	
New conduction abnormality	42/160 (26.2%)	459 (16.0%) **	217/2695 (8.0%) ***		84/438 (19.1%)
Persistent positive blood cultures	21/160 (13.1%)	413/3088 (13.4%)	251/2699 (9.0%)		
Pulmonary embolism	10/160 (5.6%)	366/3116 (11.7%) *			
Intracranial haemorrhage	5/160 (3.1%)	138/3116 (4.4%)		14/677 (2.1%)	
Vascular embolic event	54/160 (33.8%)	1429/3116 (45.8%) **	1067/2781 (38.6%)	57/677 (8.4%) ***	
Cerebral embolism	38/160 (23.8%)	633/3116 (20.3%)			
Stroke/TIA	30/160 (18.8%)	403/3116 (12.9%) *	462/2727 (17.0%)		89/438 (20.3%)
Splenic/Renal embolism/ Spondylodiscitis	14/160 (8.1%)	762/3116 (24.5%) ***			
Septic shock	33/160 (20.6%)	494/3116 (15.9%)			
***Valvular assessment:***					
Vegetation present:	134/160 (83.8%)		2406/2764 (87.0%)	605/677 (92.0%)	
AV	66/160 (41.3%)		1031/2741 (38.0%)		
MV	55/160 (34.4%)		1125/2740 (41.0%)		
TV	14/160 (8.8%)		323/2741 (12.0%)		
PV	1/160 (0.6%)		29/2739 (1.0%)		
Paravalvular abscess	31/160 (19.4%)		(15.0%)	77/677 (11.4%) **	
Prosthetic valve complication	57/160 (35.6%)		518/2781 (18.2%) ***		
Aortic valve affected	77/160 (48.1%)	(49.5%)			258/438 (58.9%) *
Mitral valve affected	65/160 (41%)	(42.0%)			
Tricuspid valve affected	21/160 (13.1%)	(11.4%)			
Pulmonal valve affected	1/160 (0.6%)	(2.4%)			
Multiple valves affected	31/160 (19.3%)	(18.2%)			
Valvular assessment:					

Overview of IE complications and valvular assessments reported in the five studies. Cell markings with asterisks represents the statistical significance levels of the two-sample Pearson’s chi-squared tests for the observed proportions. Reported significance levels for pairwise comparison between OLV Hospital and each of the four other registries individually are the following: no asterisks marking given for *p* >= 0.05, * for *p* < 0.05, ** for *p* < 0.01 and *** for *p* < 0.001.
